# Ultrasonographic and Radiographic Evaluation of Osteoarthritic Changes in the Temporomandibular Joint

**DOI:** 10.3390/diagnostics15091160

**Published:** 2025-05-02

**Authors:** Didem Dumanlı, Çiğdem Şeker

**Affiliations:** Department of Dentomaxillofacial Radiology, Faculty of Dentistry, Zonguldak Bülent Ecevit University, Zonguldak 67600, Turkey; didem.dumanli@hotmail.com

**Keywords:** cortical bone, degeneration, panoramic radiography, temporomandibular joint, tomography, ultrasonography

## Abstract

**Background/Objectives**: This study aims to determine the sensitivity, specificity, positive predictive value, and negative predictive value by comparing ultrasonography and panoramic radiography with the gold standard cone beam computed tomography in the diagnosis of osteoarthritic changes in the temporomandibular joint (TMJ) and to determine the distribution of these degenerations in terms of age and gender. **Methods**: In the study, cone beam computed tomography (CBCT), panoramic radiography, and ultrasonography (USG) images of 143 patients who applied to the Dentomaxillofacial Radiology Department of the Faculty of Dentistry of Zonguldak Bülent Ecevit University with complaints of TMJ were retrospectively examined. **Results**: As a result of the analysis, the average age of the patients included in the study was found to be 50.3 ± 14.4. The incidence of degenerative changes was higher in females than in males. The most common degenerative change in both genders was found to be flattening. Of the 143 patients’ degenerative changes detected on CBCT, 135 (94.4%) were detected on panoramic radiography and 124 (86.7%) were detected on USG. **Conclusions**: The sensitivity rates of ultrasound and panoramic radiography were found to be lower than those of CBCT in detecting degenerative changes.

## 1. Introduction

The temporomandibular joint (TMJ) enables the fulfillment of functions such as speech, chewing, and swallowing and is characterized by a complex anatomy, physiology, and biomechanics. Temporomandibular joint disorders (TMDs) are common musculoskeletal conditions that affect the maxillofacial region and are caused by factors such as stress, trauma, parafunctional habits, hormonal changes, and malocclusion, which can often be accompanied by pain. A review of the current literature indicates that temporomandibular disorders affect approximately 34% of the population, with epidemiological characteristics varying across different continents [1,2,3]. Because TMDs are sometimes asymptomatic, their diagnosis requires thorough anamnesis, clinical examination, and radiographic examination [1,2]. Like other joints, the TMJ undergoes continuous remodeling due to functional and/or parafunctional use. Functional remodeling does not lead to significant changes in mechanical functions, whereas dysfunctional remodeling, caused by factors such as parafunctional habits, stress, and inflammatory conditions, leads to degenerative changes. Such changes are more frequently observed with advancing age [4]. According to Okeson’s classification [2], TMDs are divided into three main groups: derangements of condyle–disc disorders, structural incompatibility of the articular surfaces, and inflammatory disorders of the TMJ. Osteoarthritis is an inflammation-related disease that causes degenerative changes in the mandibular condyle and glenoid fossa. Diagnosis is typically made through clinical examination and radiographic imaging [2].

Various methods can be used to visualize degenerative changes radiographically, including conventional radiography, computed tomography (CT), cone beam computed tomography (CBCT), magnetic resonance imaging (MRI), and ultrasonography (USG) [5]. Conventional radiography is useful in visualizing advanced hard tissue degeneration but is insufficient for evaluating degenerative changes at the initial stage [6]. CBCT has been widely adopted in dentistry and is now considered the gold standard for the evaluation of hard TMJ tissue components. However, it has disadvantages such as the use of ionizing radiation and the repeated exposure of the patient to radiation due to the need for multiple follow-ups. Therefore, the feasibility of USG, which can visualize both hard and soft tissue components, in the visualization of degenerative changes is being investigated [4,7].

Diagnostic accuracy studies on ultrasonographic (USG) imaging of osteoarthritic changes in the temporomandibular joint (TMJ) remain limited. Expanding the use of USG—traditionally employed for soft tissue imaging—to the evaluation of hard tissue structures such as the TMJ may enhance clinicians’ knowledge and experience in this area. As a result, patient exposure to ionizing radiation can be minimized, and USG, as a rapid and repeatable imaging modality, may provide supportive imaging findings that correlate with clinical assessments.

This study aimed to compare the sensitivity and specificity of CBCT, panoramic radiography, and USG in the detection of osteoarthritic changes in the TMJ. It also aimed to determine the distribution of osteoarthritic changes in the condyle by gender and age.

## 2. Materials and Methods

### 2.1. Sample Size

This retrospective study analyzed panoramic radiography, CBCT, and USG images of 143 patients (286 bilateral TMJs) aged 18–77 years presenting to the Department of Oral and Maxillofacial Radiology of Zonguldak Bülent Ecevit University between January and November 2022. The selected patients presented with TMJ-related issues, including pain, tinnitus, crepitus, scraping, grating, grinding symptoms, episodic pain, chronic motion restriction, functional difficulties, and limited mouth opening. Patients with missing CBCT, panoramic radiography, or USG images and patients who were under 18 years of age, were traumatized for any reason, or had any pathologic lesion in the region of interest, any syndrome, or any congenital anomaly were excluded from the study.

Panoramic radiographs were acquired during the patients’ initial hospital visits as part of routine examinations. USG images were acquired to evaluate the soft tissue components of the TMJ and masticatory muscles. CBCT images were acquired to assess the joint space, the shape of the glenoid fossa and articular eminence, and osteoarthritic changes in the condyle. The CBCT images were obtained in the Department of Dentomaxillofacial Radiology or from other clinics upon request. These patients were selected after evaluating the records and images of 1450 patients obtained at different times using all three imaging modalities. A total of 286 bilateral TMJ images from 143 patients were analyzed in this study. TMJ images without any signs of degenerative changes served as the control group. Among the 286 images evaluated, 41 represented healthy joints, while 245 exhibited degenerative changes. The patients’ images were compiled and archived anonymously. CBCT images of the mandibular condyle (one of the hard tissue components of the TMJ) showing at least one of six different degenerative changes (erosion, osteophytes, flattening, subcortical cysts, sclerosis, and loose bodies) in one or more of the medial, lateral, superior, anterior, and posterior regions were examined. Panoramic radiography and bilateral USG images were compared with CBCT, the gold standard.

### 2.2. Radiographic Examinations

Panoramic radiographs were obtained using a Veraview IC5 HD (J Morita, Kyoto, Japan) device at 60–70 kVp and 4–7.5 mA at doses for adults. CBCT images were obtained using a Veraviewepocs 3D R100/F40 (J Morita, Kyoto, Japan) device in TMJ mode in a 4 × 8 cm field of view at 90 kVp and 5 mA with a 0.125 mm^3^ voxel size. The CBCT images were evaluated using i-Dixel 2.0 software (J. Morita, Osaka, Japan).

Radiographically visible degenerative changes in the TMJ were defined as follows: Flattening was defined as a flat bone contour deviating from a convex form [4]. An osteophyte was defined as a marginal bony prominence on the mandibular condyle [4,8]. Erosion was defined as compromised integrity of the cortical bone and adjacent subcortical bone [9]. A subcortical cyst was defined as a round radiolucent area under the cortical bone or deep in the trabecular bone. Although not a true cyst, a subcortical cyst is visualized as a cystic area due to the loss of trabeculation in the bone [4]. Sclerosis was defined as an area of increased cortical bone density extending into the bone marrow [10]. Finally, loose bodies were defined as free bodies in the joint cavity separated from the osteophyte surfaces [4].

### 2.3. Ultrasonographic Examinations

USG images were obtained with a MyLab Twice (Esaote, Genoa, Italy) instrument using an extraoral linear probe in the frequency range of 4–13 MHz and analyzed using special ultrasound imaging software (MyLab Desk; Esaote, Genoa, Italy).

A standard protocol was followed for ultrasonography. All imaging was performed by a dentomaxillofacial radiology specialist with 4 years of experience. The probe was placed in the preauricular area just below the zygomatic arch. The condyle, joint space, articular disc, and temporal bone contours were evaluated as anatomical reference points. During USG imaging, the mandibular condyle surfaces were examined with the probe positioned transversely and the mouth closed.

The mandibular condyle surface and articular eminence were visualized as a hyperechogenic band, and the articular disc was visualized as a thin hypoechogenic band between the two striations [1,11,12]. Static and dynamic images of the joint were obtained and saved in TIFF and AVI formats.

Ultrasonographically visible degenerative changes in the TMJ were defined as follows: Flattening was defined as a flattened mandibular condyle due to a disruption to its concave structure [13]. In evaluating flattening on USG, several factors have been taken into consideration to determine whether it is an anatomical variant or a pathology. In anatomical variants, symmetry is usually preserved on the joint surfaces, the echogenicity of the condyle and articular disc is within normal limits, and there are no pathological findings, such as edema or increased synovial fluid in the surrounding tissues. Pathological flattening usually indicates an irregularity or asymmetry of the condylar surface. USG can detect a displacement or abnormal position of the articular disc. Since the patients included in this study had complaints, only pathological flattening was evaluated. Erosion was defined as an interruption to the echogenicity of the cortical surface [9,12]. A subcortical cyst was defined as posterior acoustic enhancement with hypoechogenicity from the subcortical area and/or increased echogenicity in the posterior wall. An osteophyte was described as a protrusion with hyperechogenicity on the mandibular condyle [13]. Sclerosis was defined as an increased echogenicity of the mandibular condyle. Due to the limited literature on ultrasonographic imaging of sclerosis, Pihut et al.’s study was used as a reference [10]. Finally, loose bodies were defined as hyperechogenicity visualized within the joint capsule outside the borders of the mandibular condyle [13].

### 2.4. Assessment of Radiographic Images

**Figure 1:**
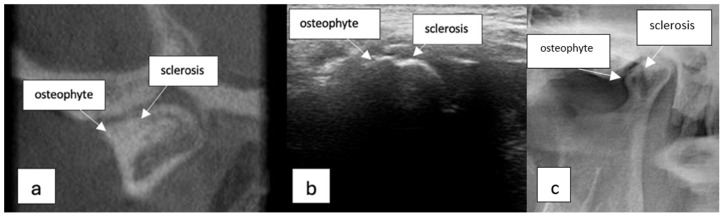
TMJ osteophyte and sclerosis. (**a**) CBCT image (sagittal view), (**b**) USG image, and (**c**) panoramic radiography image.

**Figure 2:**
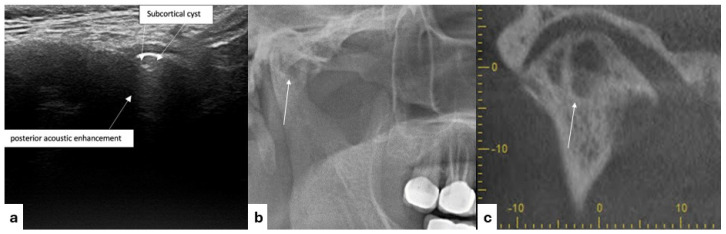
Ultrasonographic (**a**) and radiographic images of a subcortical cyst (**b**,**c**).

**Figure 3:**
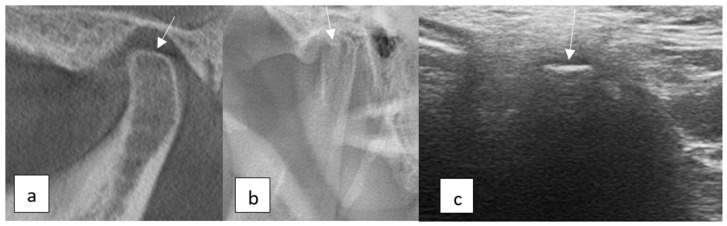
TMJ flattening: (**a**) CBCT image showing flattening in the superior surface of the condyle (sagittal view), (**b**) panoramic radiography image, and (**c**) USG image.

**Figure 4:**
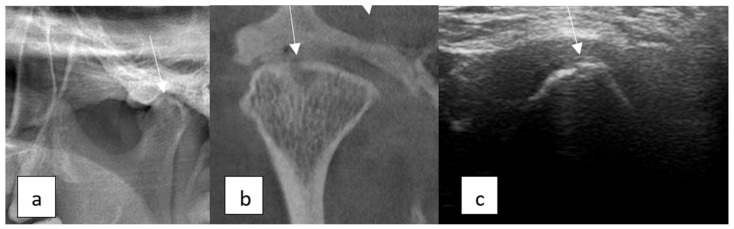
TMJ erosion: (**a**) panoramic radiography image, (**b**) CBCT image showing erosion in the superior surface of the condyle (sagittal view), and (**c**) USG image.

**Figure 5:**
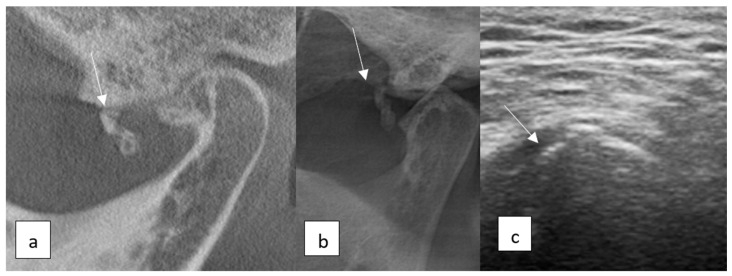
Loose body of TMJ: CBCT image showing loose body (white arrow) and osteophyte on the anterior surface of the condyle (**a**), panoramic radiography image (**b**), and USG image (**c**).

The images were evaluated by a dentomaxillofacial radiology specialist and a dentomaxillofacial radiology research assistant at two time points one month apart. The first evaluation was conducted one month after the data were archived to ensure that the observers had forgotten the images. Thus, blind assessment was performed. The presence or absence of degenerative changes in the TMJ was determined using CBCT images as the gold standard. Disagreements between the two observers were resolved through discussion. To ensure consistency, all images were displayed on the same 23-inch color LCD monitor (RadiForce MS230W; EIZO, Ishikawa, Japan) (Figure 1, Figure 2, Figure 3, Figure 4 and Figure 5).

To evaluate interobserver agreement, 10% of the images were randomly selected and examined twice by the two observers with a two-week interval. Agreement was quantified using the Kappa statistics. The results showed very good agreement: 0.948 for CBCT (*p* < 0.001), 0.85 for USG (*p* < 0.001), and 0.91 for panoramic radiography (*p* < 0.001).

### 2.5. Statistical Analysis

In descriptive statistics, continuous variables were expressed as means and standard deviations, whereas categorical variables were expressed as frequencies and percentages. The chi-squared test was used in the analysis of independent qualitative data. When the conditions for the chi-squared test were not met, Fischer’s exact test was used. Agreement between qualitative data was assessed using the Kappa statistic. Values of *p* < 0.05 were considered statistically significant. The analyses were performed using IBM SPSS Statistics 28.0 (IBM, Armonk, NY, USA).

To determine statistically significant differences in the rate of degenerative changes by age, the patients were divided into two groups: 50 years and younger (50 years old incl.) and over 50 years.

#### Power Analysis

The sample size was calculated using G*Power 3.1 software, based on a 5% margin of error (95% confidence level), 80% statistical power, and a standard effect size of 0.33. Power analysis indicated that at least 143 cases needed to be included in the study.

These parameters are widely accepted and supported by the literature in biomedical research. An 80% power increases the study’s ability to detect meaningful differences, while an effect size of 0.33 allows for the identification of clinically relevant effects. In accordance with contemporary methodological recommendations, the results were not only reported using *p*-values but were also supplemented with effect size calculations for each statistical analysis. Effect sizes were categorized as small (0.2), medium (0.5), or large (>0.8), following Cohen’s conventions. However, considering that thresholds of 0.10, 0.30, and 0.70 are specifically recommended for studies on the temporomandibular joint (TMJ) and masticatory muscles, these adjusted values were adopted as reference points for interpreting the results in these studies [14,15]. In interpreting the findings, clinical relevance was attributed exclusively to medium and large effect sizes. Small effect sizes were explicitly considered clinically insignificant and treated as such in the discussion of the results.

## 3. Results

Among the 143 patients, 110 (77%) were female, and 33 (23%) were male. The mean age of the patients was 50.3 ± 14.4 years. The age distribution of the patients included in the study was as follows: 3 patients were in the 18–20 age range, 12 in the 21–30 range, 25 in the 31–40 range, 33 in the 41–50 range, 31 in the 51–60 range, and 39 in the 61–77 range. The rates of degenerative changes in these two age groups detected using each of the three imaging modalities are shown in Table 1. The visibility of erosions in the group with an age of over 50 years was significantly higher on CBCT and USG than on panoramic radiography (Table 1).

The distribution of the localization of degenerative changes on CBCT is presented in Table 2. Flattening was the most frequently detected degenerative change among both male and female patients in all three modalities. There was no statistically significant difference between male and female patients in terms of degenerative changes on CBCT, USG, or panoramic radiography (Table 1 and Table 3).

The detection of degenerative changes showed almost perfect agreement between the first and second intra-observer evaluations on panoramic radiography and USG (Table 4). However, the detection rate was significantly lower on USG than on panoramic radiography and CBCT. Nevertheless, the rate of detecting erosion and flattening on USG was similar to that obtained from panoramic radiography (Table 5). A receiver operating characteristic curve analysis showed that the rate of detecting loose bodies and subcortical cysts on USG was quite low (Table 6).

## 4. Discussion

Degenerative changes can sometimes be asymptomatic. Accurate diagnosis requires a thorough history, clinical examination, and, when necessary, imaging. Panoramic radiography is the first-line modality in routine examinations. CBCT is very effective in detecting degenerative changes in hard TMJ tissue, whereas MRI and USG are used to image the masticatory muscles and the soft tissue components of the TMJ [1,2,3,4,5].

Prevalence studies involving different ethnic groups have reported flattening, erosion, and osteophytes as the most frequently observed degenerative changes in the TMJ [4,16,17,18]. The results of this study are consistent with these findings. Moreover, as in other studies, degenerative changes were observed in female patients almost three times as frequently as in male patients. However, no significant differences in degeneration types were found between female and male patients. The mean age of the patients in this study was higher than in other studies using CBCT [4,16,17]. This may be because all patients included in this study exhibited degenerative changes.

Flattening is the first adaptive response of the TMJ to excessive occlusal loads [19]. This likely explains why it is the most common degenerative change. Osteophytes are bony protrusions formed by expanding the surface area of the TMJ to develop tolerance to excessive occlusal forces. They usually occur in the advanced stages of degenerative changes. Erosion is the initial stage of degenerative changes [20].

CBCT generally demonstrates higher accuracy and sensitivity in detecting bone changes in the TMJ and mandibular condyles than other imaging modalities, such as conventional tomography, CT, and panoramic radiography. Studies have shown that CBCT is effective in identifying cortical erosions and osseous defects, particularly small ones, although its accuracy is influenced by the size of the defect. While the performance of CBCT is similar to that of CT, the former offers the advantage of lower radiation doses. Moreover, CBCT-based 3D models have proven superior in both qualitative and quantitative assessments of condylar morphology changes. However, many studies have overlooked the impact of soft tissues, which may limit the clinical applicability of their findings. Thus, the diagnostic accuracy of CBCT varies depending not only on defect size but also on the imaging protocol used [21].

Studies evaluating TMDs using USG have mostly focused on the articular disc and have generally compared the performance of USG to that of MRI [1,22]. In a comprehensive comparison of the accuracy of MRI and USG in detecting TMDs, Zaman et al. found that MRI had a lower risk of false negatives and misdiagnoses than USG and was superior in terms of diagnostic efficacy. Nevertheless, the authors noted that USG, as a less invasive technique, could play an important role in the initial evaluation, with MRI serving as a confirmatory method [20]. Similarly, our study results indicate that USG may be useful in the preliminary evaluation of TMDs.

As previously noted, panoramic radiography can be used to detect large erosions and osteophytes in the TMJ [23]. Patient positioning is a critical factor in panoramic radiography. If the patient’s head is tilted backward, the condyle flattens and may resemble osteophytes. If the head is tilted forward, the condyle may appear sclerotic. In a panoramic radiography study using cadaveric skulls, Fallon et al. found that it was impossible to determine the condyle morphology in an ideal way due to radiographic variations caused by differences in condylar angulation [24]. Another disadvantage of panoramic radiography is that it cannot visualize the TMJ well because the skull base and zygomatic arches are superimposed [24]. This situation explains why the sensitivity of panoramic radiography is lower than that of CBCT in our study.

In a study evaluating various degenerative TMDs using MRI, CT, and panoramic radiography, the sensitivity of panoramic radiography varied between 12% and 33%, that of MRI ranged from 32% to 70%, and that of CT ranged from 99% to 100%. Panoramic radiographs demonstrated low sensitivity but excellent specificity in detecting signs of subcortical cysts, erosion, osteophytes, and sclerosis [25]. Hechler et al. conducted a systematic review of 19 studies comparing MRI and USG and found that the overall sensitivity of high-resolution USG in assessing condylar bone destruction, when compared to MRI, ranged from 36.8% to 87%, with specificity ranging from 20% to 83.1%, and accuracy in diagnosing bone destruction ranging from 55.9% to 76% [26].

A previous study evaluating TMDs using USG and CBCT showed that USG had high sensitivity (86%) and negative predictive value (89%) in identifying normal anatomical features, making it a valuable tool for excluding TMJ abnormalities. However, it had limited sensitivity in detecting significant degenerative changes, such as osteophytes (18%) and erosion (14%) [27]. In our study, we determined that USG had higher sensitivity for erosion and osteophytes.

Previous studies evaluating the TMJ using USG have used frequencies ranging from 7.5 to 20 MHz [24]. The use of probes with an increasing frequency (10 MHz and higher) has been shown to result in 60–90% higher sensitivity, depending on the increase in frequency [26,28,29,30]. Erosion, loose bodies, and osteophytes appear similar on USG [13], affecting its sensitivity. It has been reported that among the degenerative changes on the posterior, anterior, medial, lateral, and superior surfaces of the mandibular condyle visible on CBCT, only those on the superior and anterior surfaces can be clearly seen on USG [9,12]. The reason is thought to be that the lateral surface of the mandibular condyle prevents sound waves from passing to the medial surface [9]. Moreover, the accuracy of USG examination depends heavily on the operator’s skills and experience [31]. Differences in opinion between researchers may be due to a lack of standardization in examination procedures, the choice of probe, the probe settings used, and whether the mouth is open or closed. High-frequency USG using a probe with a frequency equal to or higher than 12 MHz provides better visualization of the TMJ [31,32].

This study has several limitations. The study sample consisted only of individuals with complaints, limiting the generalizability of the findings to the overall TMJ patient population.

Similarly, the fact that this was a single-center study limits the generalizability of the results. Moreover, the imbalanced sex distribution (77% women and 23% men) may have influenced the findings. Furthermore, USG, being operator-dependent, may have affected the reproducibility of the results, and the evaluations were made by only two operators. The retrospective nature of the study may also have affected the evaluation of USG data. Finally, a detailed analysis of clinical symptoms and their correlations with imaging findings was not performed. Future prospective studies with more balanced samples are needed to address these limitations.

Regarding USG protocols for TMJ evaluations, the use of high-frequency probes is recommended. Moreover, examining patients with their mouths open, closed, and while performing dynamic movements can provide a better evaluation of degenerative joint changes. Since our study was retrospective and required standardization for evaluation, only USG images in the mouth closed position were included in the study. In follow-ups, USG can be used as a bridging modality between MRI or CBCT protocols. This allows for a quick assessment before resorting to modalities that are more time-consuming and expensive and involve radiation exposure. Finally, more studies on the visualization of osteoarthritic changes in the TMJ using USG. Finally, more studies are needed on the imaging of osteoarthritis changes in the TMJ using USG. The findings and images obtained will provide a reference for operator evaluation.

## 5. Conclusions

CBCT remains the most effective imaging modality for detecting degenerative changes in the TMJ due to its ability to provide detailed visualization of hard tissue. While panoramic radiography is limited by its two-dimensional nature and the superposition of anatomical structures, it offers a cost-effective and accessible initial diagnostic tool. On the other hand, USG provides a radiation-free alternative that can be used in the evaluation of both hard and soft tissues. However, its sensitivity and specificity, especially in detecting subtle degenerative changes such as loose bodies and subcortical cysts, are comparatively low.

## Figures and Tables

**Table 1:** TMJ degenerative changes detected on CBCT, USG, and panoramic radiography by age group.

Degenerative Change	Present (+)/Absent (−)	Age ≤50		Age >50		*p*	Effect Size
		*n*	%	*n*	%		
Erosion							
CBCT	−	103	70.5	76	52.1	0.003	0.30
	+	43	29.5	64	43.8		
USG	−	127	87	106	72.6	0.014	0.27
	+	19	13	34	23.3		
Panoramic radiography	−	121	82.9	109	74.7	0.285	0.10
	+	25	17.1	31	21.2		
Osteophytes							
CBCT	−	105	71.9	93	63.7	0.315	0.09
	+	41	28.1	47	32.2		
USG	−	133	91.1	117	80.1	0.055	0.21
	+	13	8.9	23	15.8		
Panoramic radiography	−	118	80.8	115	78.8	0.774	0.05
	+	28	19.2	25	17.1		
Flattening							
CBCT	−	50	34.2	53	36.3	0.525	0.13
	+	96	65.8	87	59.6		
USG	−	69	47.3	81	55.5	0.073	0.25
	+	77	52.7	59	40.4		
Panoramic radiography	−	63	43.2	60	41.1	0.960	0.04
	+	83	56.8	80	54.8		
Loose bodies							
CBCT	−	142	97.3	131	89.7	0.134	0.17
	+	4	2.7	9	6.2		
USG	−	145	99.3	138	94.5	0.616	0.07
	+	1	0.7	2	1.4		
Panoramic radiography	−	143	97.9	136	93.2	0.661	0.04
	+	3	2.1	4	2.7		
Subcortical cysts							
CBCT	−	143	97.9	137	93.8	0.959	0.21
	+	3	2.1	3	2.1		
USG	−	146	100	139	95.2	0.490	0.12
	+	0	0	1	0.7		
Panoramic radiography	−	143	97.9	138	94.5	0.686	0.18
	+	3	2.1	2	1.4		
Sclerosis							
CBCT	−	122	83.6	115	78.8	0.750	0.12
	+	24	16.4	25	17.1		
USG	−	136	93.2	125	85.6	0.247	0.13
	+	10	6.8	15	10.3		
Panoramic radiography	−	131	89.7	123	84.2	0.616	0.04
	+	15	10.3	17	11.6		

The *p* values in bold indicate statistically significant differences.

**Table 2:** Distribution of the localization of TMJ degenerative changes on CBCT.

Localization	Erosion		Osteophytes		Flattening		Sclerosis		Subcortical Cysts		Loose Bodies		Total	
	*n*	%	*n*	%	*n*	%	*n*	%	*n*	%	*n*	%	*n*	%
Right	28	28	27	27	22	22	17	17	3	3	3	3	100	100
Left	33	30.6	33	30.6	13	12	18	16.7	3	2.7	8	7.4	108	100
Bilateral	23	19.3	14	11.8	74	62.2	7	5.9	0	0	1	0.8	119	100
Total	84	25.7	74	22.7	109	33.4	42	12.8	6	1.8	12	3.6	327	100

**Table 3:** TMJ degenerative changes in male and female patients.

Degenerative Change	Present (+)/ Absent (−)	Female		Male		*p*	Effect Size
		*n*	%	*n*	%		
Erosion							
CBCT	−	142	64.5	37	56.1	0.212	0.17
	+	78	35.5	29	43.9		
USG	−	180	81.8	53	80.3	0.781	0.04
	+	40	18.2	13	19.7		
Panoramic radiography	−	177	80.5	53	80.3	0.978	0.01
	+	43	19.5	13	19.7		
Osteophytes							
CBCT	−	151	68.6	47	71.2	0.691	0.06
	+	69	31.4	19	28.8		
USG	−	190	86.4	60	90.9	0.329	0.14
	+	30	13.6	6	9.1		
Panoramic radiography	−	180	81.8	53	80.3	0.781	0.04
	+	40	18.2	13	19.7		
Flattening							
CBCT	−	79	35.9	24	36.4	0.946	0.01
	+	141	64.1	42	63.6		
USG	−	116	52.7	34	51.5	0.863	0.02
	+	104	47.3	32	48.5		
Panoramic radiography	−	96	43.6	27	40.9	0.695	0.05
	+	124	56.4	39	59.1		
Loose bodies							
CBCT	−	210	95.5	63	95.5	1	-
	+	10	4.5	3	4.5		
USG	−	218	99.1	65	98.5	0.546	0.06
	+	2	0.9	1	1.5		
Panoramic radiography	−	215	97.7	64	97	0.664	0.04
	+	5	2.3	2	3		
Subcortical cysts							
CBCT	−	215	97.7	65	98.5	1	0.06
	+	5	2.3	1	1.5		
USG	−	219	99.5	66	100	1	0.10
	+	1	0.5	0	0		
Panoramic radiography	−	215	97.7	66	100	0.593	0.22
	+	5	2.3	0	0		
Sclerosis							
CBCT	−	179	81.4	58	87.9	0.218	0.18
	+	41	18.6	8	12.1		
USG	−	199	90.5	62	93.9	0.379	0.13
	+	21	9.5	4	6.1		
Panoramic radiography	−	196	89.1	58	87.9	0.784	0.04
	+	24	10.9	8	12.1		

**Table 4:** Intra-observer agreement in detecting TMJ degenerative changes on USG and panoramic radiography.

Degenerative Change	Kappa	
	USG	Panoramic Radiography
Erosion	0.940	0.989
Osteophytes	0.933	0.988
Flattening	0.979	0.979
Loose bodies	1	0.921
Subcortical cysts	1	1
Sclerosis	0.956	0.948

**Table 5:** Sensitivity, positive predictive value, specificity, negative predictive value, and diagnostic accuracy of USG and panoramic radiography in detecting TMJ degenerative changes.

Degenerative Change	Sensitivity (%)	PPV (%)	Specificity (%)	NPV (%)	Diagnostic Accuracy (%)
Erosion					
USG	45	91	97	75	78
Panoramic radiography	52	100	100	78	82
Osteophytes					
USG	39	94	99	78	80
Panoramic radiography	60	100	100	85	88
Flattening					
USG	73	99	98	67	82
Panoramic radiography	89	100	100	84	93
Loose bodies					
USG	15	67	99	96	96
Panoramic radiography	46	86	99	98	97
Subcortical cysts					
USG	17	100	100	98	98
Panoramic radiography	61	60	99	99	98
Sclerosis					
USG	45	88	99	90	90
Panoramic radiography	63	97	100	93	93

PPV: positive predictive value; NPV: negative predictive value.

**Table 6:** Areas under the curve (ROC) of panoramic radiography and USG compared to the gold standard (CBCT).

Degenerative Change	AUC (ROC)	*p*
Erosion		
USG	0.710	0.000
Panoramic radiography	0.762	0.000
Osteophytes		
USG	0.688	0.000
Panoramic radiography	0.801	0.000
Flattening		
USG	0.856	0.000
Panoramic radiography	0.945	0.000
Loose bodies		
USG	0.575	0.360
Panoramic radiography	0.729	0.005
Subcortical cysts		
USG	0.583	0.485
Panoramic radiography	0.746	0.039
Sclerosis		
USG	0.718	0.000
Panoramic radiography	0.814	0.000

The *p* values in bold are statistically significant. AUC: area under the curve.

## Data Availability

Most of the data generated or analyzed are included in the article. The remaining datasets used and/or analyzed during the current study are available from the corresponding author upon request.

## Abbreviations

The following abbreviations are used in this manuscript:

TMJ	Temporomandibular Joint
TMDs	Temporomandibular Joint Disorders
CBCT	Cone Beam Computed Tomography
USG	Ultrasonography
CT	Computed Tomography
MRI	Magnetic Resonance Imaging
